# Intrinsic motivation between face-to-face and blended learning in surgical clinical education

**DOI:** 10.12669/pjms.40.5.1048

**Published:** 2024

**Authors:** Masood Jawaid, Zubia Masood, Nazish Imran

**Affiliations:** 1 Masood Jawaid Director Medical Affairs, PharmEvo, Karachi, Pakistan; 2 Dr. Zubia Masood Associate Professor, Dept. of Surgery, Baqai Medical University, Karachi, Pakistan; 3 Prof. Nazish Imran Mayo Hospital and Kind Edward Medical University, Lahore, Pakistan

**Keywords:** Blended learning, Face-to-face learning, Intrinsic motivation inventory, Medical education, Medical students, Surgical teaching

## Abstract

**Objective:**

The variability and opportunistic nature of surgical clinical education is the main problem for effective teaching and training of medical students. Incorporating online mediums including discussion forums, interactive videos/scenarios, static pages, and quizzes is known as blended learning (BL). This study aimed to compare the intrinsic motivation of surgical students enrolled in blended learning to those enrolled in face-to-face teaching (f2f teaching).

**Methods:**

A quasi-experimental, cross-over study was conducted in Surgical Unit-I and Surgical Unit-II of Dow University Hospital, Karachi, from March to August 2014. A total of 31 students participated and were exposed to two different teachings. For the first four weeks, Group A was posted in Surgical-I (f2f teaching) and Group B in Surgical-II (BL). Both groups were taught the same contents with the same schedule. The F2F group had clinical exposure to real patients, and small group discussions (SGDs) while The BL group students were exposed to an additional online learning component. Intrinsic Motivation Inventory (IMI) was administered at the end of four weeks and groups were swapped. Exchanged groups were again taught the same contents with the same schedule for another four weeks and IMI was administered.

**Results:**

Fifty-eight students completed IMI; 28 in f2f and 30 in BL group. There was a significant difference in all four subscales of IMI between the two groups. In three subscales, students in BL were more motivated as compared to f2f (p<0.01). Students in f2f experienced more perceived tension than in BL (p<0.048).

**Conclusion:**

This study concluded that blended surgical learning programs keep medical students more intrinsically motivated to learn. By utilizing online learning, superior educational opportunities for students can be cultivated. It can result in enhanced faculty effectiveness and efficiency as well.

## INTRODUCTION

Clinical education is the teaching and learning concentrated on and involving, patients and their problems; it forms the most integral part of medical education. Medical institutes facilitate their students with as much clinical exposure as possible early in their education and this is one of the reasons for the success of contemporary integrated curriculum.^1^ These activities, e.g. history taking from a patient and examining; augment the understanding of knowledge from the classroom, an artificial setting, to the workplace, the real world.^2^ It results in enhanced clinical reasoning skills, learning relevance, and increased student motivation.^3^,^4^

Motivation is the student’s desire to learn the course.^5^ There is evidence that it has important consequences on learning efficiency. Discrete features and situational impacts like teaching methods have been documented to have both direct as well as indirect effects on the student’s motivation for learning.^6^ The common forms of motivation include intrinsic motivation, Identified motivation, and Introjected Motivation. The most independent type of motivation, intrinsic motivation is what propels people to engage in activities out of sincere interest and satisfaction. Identified motivation is an autonomous type of motivation, yet one that is more than intrinsic motivation, even if it is an instrumental or extrinsic form of motivation. Engaging in activities due to internalized feelings of compulsion, pressure to meet norms, or self-esteem contingencies is known as introjected motivation.^7^,^8^

Surgical students can experience two forms of learning. Face-to-face (f2f) learning includes bedside teaching, small group discussions, and clinical observership in different clinical sites of a surgical hospital-like outpatient department, inpatient ward, and operation theatre. In addition to face-to-face learning, online mediums (delivered via the internet) which include discussion forums, flash interactive videos, Virtual Patients, static pages, and quizzes will be used to learn clinical skills during the same duration of time.^9^,^10^ This is known as blended learning (BL). The terms blended, hybrid, and technology-enhanced learning are often used synonymously. A blended style of clinical teaching may have the benefit of addressing some of the intricacy and distinction inherent in this type of education.^11^ The blended approach integrates online and f2f instructions to support meaningful interaction between teachers, learners, and learning content.^12^ BL has the potential to make sure that every student has a standardized learning experience in this context.

Both f2f and BL affect the intrinsic motivation of students in a separate way. In the f2f method, the ability of the teacher to engage and influence the students both verbally and non-verbally improves the learning experience and enhances their motivation. Numerous intrinsic pedagogical aspects of online learning improve students’ motivation which include increased interaction, more time utilization, provision of additional learning material, and increased student control over the speed of learning, apart from when and where students want to learn.^6^

A planned structured online experience can motivate students to become more involved with the learning material.^13^ BL provides learners with the good features of both f2f and online mediums while diminishing the undesirable aspects of each method.^14^ There is plenty of evidence available that the knowledge gained by learners in BL programs is greater than students in either f2f or online modules.^15^,^16^ We aimed to compare the intrinsic motivation of surgical students enrolled in blended learning to those enrolled in face-to-face teaching.

## METHODS

A quasi-experimental, cross-over study was conducted in Surgical Unit-I and Surgical Unit-II of Dow University Hospital, Karachi, from March to August 2014. All students of semesters V and VI, MBBS program, posted in the surgery department during that period were included. These students had a minimum of 70% of attendance during their clinical posting. Students not sitting in OSCE at the end of their clinical posting were excluded.

### Ethical Approval:

IRB approval was taken from Dow University of Health Sciences (IRB-408/DUHS/-13; dated December 12, 2013).

**Fig.1:**
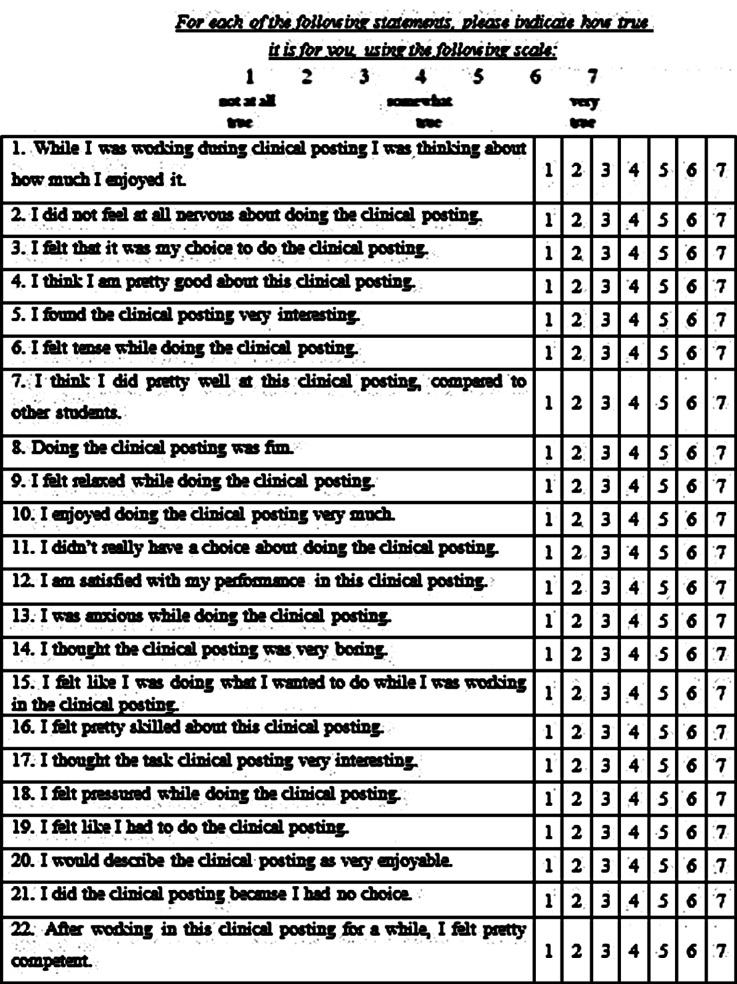
Twenty-two Item Intrinsic Motivation Inventory (IMI).

Students posted in surgery wards were divided into two groups. During the first four weeks of the study, the group posted in Surgical Unit-I was administered face-to-face teaching and the group posted in Surgical-II was administered blended learning. At the end of four weeks, a constant Objective Structured Clinical Examination of 14 stations of five minutes each was administered to both groups. An Intrinsic Motivation Inventory (IMI)^13^ was also administered to both groups (Fig.1).^16^

The face-to-face (F2F) group experienced real patient exposure in a clinical setting, small group discussions (SGDs), simulated patient sessions where students conducted mock examinations and took mock histories for formative feedback, and theatre-case observations where students learned how to scrub, gown, and glove as well as have the opportunity to help with minor procedures. Additionally, interactive flash learning materials were created for the BL group using the Sharable Content Object Reference Model (SCORM). It generated collections of digital educational resources that could be distributed among various platforms. Videos that included clickable links, hotspots, and question-answers were uploaded as SCORM packages to provide an excellent learning experience. For the BL group, flash technology (Articulate Studio) was also used to create simulated situations. They gave the students quick feedback on the correct and wrong choices, which helped them with their clinical reasoning abilities. Faculty of both surgical units are comparative in terms of faculty positions and teaching experience.

After four weeks, the groups were swapped and students of Surgical Unit-I (face-to-face teaching) were sent to Surgical Unit-II (blended learning) and vice versa. The content and schedule were again kept constant for both groups (but different from the first four weeks). At the end of four weeks, a constant OSCE of 14 stations and 22-item IMI was administered to both groups.

IMI is a twenty-two-item scale that has four subscales: perceived choice, perceived competence, interest/enjoyment, and pressure/tension. The perceived choice and perceived competence subscales are hypothesized to be the affirmative predictors of both behavioral measures and self-report of intrinsic motivation. The interest/enjoyment subscale is taken as the self-report measure of intrinsic motivation. Pressure tension is hypothesized to be an adverse predictor of intrinsic motivation.

To score IMI, the subscale score was first calculated by averaging the items scores for the items on each subscale. Items 1, 5, 8, 10, 14, 17, 20 were for interest/enjoyment. Items 4, 7, 12, 16, and 22 were for perceived competence. Items 3, 11, 15, 19, and 21 were for perceived choices. Items 2, 6, 9, 13, and 18 were for pressure/tension. Items 2, 9, 11, 14, 19 and 21 were reverse scored. A higher score would indicate more of the concept described in the subscale name. It means that a higher score on pressure/tension reveals that the person was more tense and pressured. A higher score on perceived competence tells that the person felt more competent; and so on.

### Statistical Analysis:

SPSS version 17 was used to analyze the data. Descriptive statistics were computed. Mean and standard deviation calculated for quantitative output response. Frequency percentage computed for qualitative output response. Statistical significance was taken at p <0.05.

## RESULTS

Fifty-eight students completed the IMI inventory questionnaire with 28 students in the f2f group and 30 in the blended group. Their mean ± SD age was 21.79 ± 1.69 years with female to male ratio of 2:1. The Cronbach Alpha reliability score for the Intrinsic Motivation Inventory in this study was 0.798 which showed that this research had good internal consistency. The internal consistency of the other four subscales ranged from 0.685 to 0.917 (Table-I).

**Table-I:** Reliability of the intrinsic motivation inventory.

IMI scale	Items	Cronbach’s Alpha
All items	22	0.798
Interest / enjoyment	7	0.917
Perceived competence	5	0.828
Perceived choice	5	0.756
Perceived pressure	5	0.685

There was a significant difference in all four subscales between f2f teaching and blended teaching of surgical clinical education. In all three subscales, students in the blended group were more motivated as compared to the f2f teaching group (p <0.01).

Pressure tension is hypothesized to be an adverse predictor of intrinsic motivation. Students in the f2f group experienced more perceived tension as compared to the blended group (p < 0.048). Overall, students in the blended group were more intrinsically motivated as compared to face to face group during surgical clinical education (Table-II).

**Table-II:** Intrinsic motivation comparison of Face to face and blended teaching.

Subscale	Face to face teaching n = 28		Blended Teaching n = 30		P value*

	Mean	SD	Mean	SD	
Enjoyment	28.92	8.96	38.20	6.38	<0.0001
Competence	18.67	4.50	26.30	4.32	<0.0001
Choice	19.00	6.09	24.60	5.10	<0.0001
Pressure	20.17	6.85	17.10	4.26	<0.048

* Independent t-test applied.

## DISCUSSION

The findings of our study suggest that students learning through blended teaching were more intrinsically motivated to learn and gain new knowledge as compared to the students enrolled in face-to-face learning groups during their surgical clinical posting.

This is one of the first studies from Pakistan about BL in surgical clinical education not only because of its methodological strength but also for the evidence-based utilization of the latest educational technologies in a systemic and integrated form of innovative curriculum. One study from this data about OSCE score was already published in 2021 in J Pak Med Assoc. Although the data of the study is old, its findings and implications are still very applicable to online learning.^17^

In all subscales of enjoyment, competence, and choice there is more score in the BL group while in the perceived pressure subscale which has an inverse relationship with motivation, there is less score in the blended group. The results are comparable with numerous studies of motivation in online and blended learning.^18^,^19^ Undergraduate as well as postgraduate online students compared to on-campus students were also found to be more intrinsically motivated in comparative studies.^20^-^22^ The reasons for more motivation in online and blended courses are numerous. In traditional instructor-led classroom learning, less learner control, passive learning, and limited instructional material availability are likely to result in less motivation to learn. On the other hand, better learner control over the pace of learning/ instructions, active engagement of learners & access to a vast variety of instructional material may facilitate and enhance motivation to learn in Web based/e- learning. Literature suggests that compared to traditional instructor-led methods, e-learners gain faster and better knowledge and skills.^13^

The benefits of online and traditional face-to-face teaching are maximized in blended learning. It encompasses all adult learning theory principles i.e. being learner-centered, self-directed, and enabling the learners responsible for their learning.^23^ BL relies more heavily on technology and also needs more time commitment than classroom learning. There is robust support for a positive relationship between learning motivation and BL.^24^ Learners in BL courses were more motivated to learn, achieved higher course grades, and engaged in more metacognition than students in classroom learning.^6^ The main reasons appear to be more learner control over when and where to learn as well as more tools available to facilitate learning. It has been emphasized that to increase motivation to learn, course designers should use both synchronous and asynchronous knowledge to supplement face-to-face interactions and ensure a high level of transactional presence of an instructor. All these factors appear to increase learner’s intrinsic motivation 25

## CONCLUSION

It is likely to attain an enhancement in motivation and performance along with great acceptance of students with a blended surgical learning program. The results showed that the BL methods might be superior in comparison to face-to-face teaching alone, even in the setting of a skill-based curriculum like surgery. Online learning modalities have a potential for use in surgical education as students and trainees face significant hurdles in their training variability of exposure, less time in busy clinical settings, and less personalized formative feedback to name a few. By utilizing online learning, superior educational opportunities for students can be cultivated. It can result in enhanced faculty effectiveness and efficiency as well. This potential of e-learning undertakes a certain level of institutional inclination both in human and infrastructural resources which is not always extant in all places.

### Authors’ Contributions:

**MJ:** Conceived the idea, curriculum development, synopsis writing, data collection and analysis, intellectual contribution to the paper, and approval of the final version to be published.

**ZM:** Study design, data collection and analysis, intellectual contribution to the paper, and approval of final version to be published.

**NI:** First draft writing, literature review, and approval of final version to be published.
